# A Rapid, Field‐Deployable Diagnostic Platform for Getah Virus Based on RT‐RAA and CRISPR EsCas13d

**DOI:** 10.1111/1751-7915.70429

**Published:** 2026-08-09

**Authors:** Liu Baoling, Shao Lina, Xu Tong, Jia Qinrui, Zhu Ling, Xu Zhiwen

**Affiliations:** ^1^ College of Veterinary Medicine Sichuan Agricultural University Chengdu Sichuan China

**Keywords:** CRISPR‐EsCas13d, Getah virus, reverse transcriptional recombinase‐aided amplification, visual detection assay

## Abstract

The emerging zoonotic Getah virus (GETV) poses an increasing threat to both animal and human health, underscoring the need for rapid, sensitive and field‐deployable diagnostic tools. In this study, we developed and optimized a rapid, one‐step, visual detection (ROSVD) platform for GETV by integrating reverse transcription recombinase‐aided amplification (RT‐RAA) with CRISPR‐EsCas13d‐mediated collateral RNA cleavage. Notably, the ROSVD assay uses a simplified sample‐preparation strategy based on rapid nucleic acid release, eliminating the need for conventional nucleic acid extraction and purification. The entire workflow, including amplification and detection, is completed within 30 min at 37°C or ambient temperature (25°C) without specialized instrumentation. Detection results can be visualized directly under ultraviolet light or with a lateral flow assay. At 37°C, the ROSVD assay achieved sensitivity comparable to RT‐qPCR, and evaluation of clinical specimens showed 100% concordance with RT‐qPCR results. Collectively, these findings demonstrate that ROSVD is a rapid, sensitive, cost‐effective and instrument‐independent diagnostic platform, providing a practical solution for on‐site surveillance of GETV and a versatile framework for the detection of other emerging RNA pathogens.

## Graphical abstract

This study developed a rapid, one‐step, visual detection platform termed ROSVD by integrating RT‐RAA with CRISPR–EsCas13d, enabling simplified sample preparation, high‐sensitivity, on‐site detection of GETV within 30 min without the need for sophisticated equipment. The assay demonstrated high concordance with RT‐qPCR in clinical validation and provides a practical and cost‐effective solution for the rapid diagnosis of emerging animal and human RNA viruses.

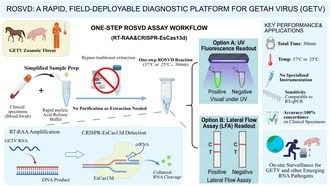

## 1 Introduction

Zoonotic viruses remain a major global concern. The outbreak of severe acute respiratory syndrome coronavirus 2 (SARS‐CoV‐2) has not only demonstrated the devastating potential of zoonotic pathogens but also underscored the critical importance of establishing robust systems for pathogen detection and early warning (Munir et al. 2020). Detection technologies constitute the cornerstone of animal disease prevention and control, supporting early warning, precise diagnosis and effective intervention against infectious diseases (Vidic et al. 2017). However, conventional high‐sensitivity methods, such as RT‐qPCR and PCR, rely on sophisticated instrumentation and trained personnel, which limits their utility for rapid, on‐site testing in clinical or field settings. In response, isothermal amplification technologies have emerged as practical alternatives. Methods including LAMP, RPA and RAA enable efficient nucleic acid amplification at constant or ambient temperatures with minimal equipment requirements, while their reverse transcription–coupled derivatives (RT‐LAMP, RT‐RPA and RT‐RAA) further extend these systems to RNA pathogen detection (Garrido‐Maestu and Prado 2022; Ghosh et al. 2022; Mao et al. 2023). However, amplification‐only assays may still suffer from non‐specific amplification when applied to complex clinical samples. These limitations have prompted increasing interest in CRISPR‐based diagnostic strategies that can enhance detection specificity while maintaining operational simplicity. Unlike amplification‐only assays, CRISPR‐based detection introduces an additional sequence‐recognition step following nucleic acid amplification (Gootenberg et al. 2017). Guided by a target‐specific CRISPR RNA (crRNA), Cas effectors are activated only upon precise hybridization with the intended amplicon, after which their collateral nuclease activity indiscriminately cleaves labelled reporter molecules to generate a detectable signal. Consequently, the crRNA‐guided target recognition that precedes collateral cleavage functions as a sequence‐specific molecular verification step, ensuring that reporter cleavage occurs only after correct target recognition and thereby substantially reducing false‐positive signals arising from non‐specific amplification (Kellner et al. 2019; Kaminski et al. 2021).

In recent years, CRISPR‐based detection methods integrated with isothermal amplification have attracted considerable attention due to their high sensitivity and specificity (Bi et al. 2025). SHERLOCK was initially introduced in 2017 as a CRISPR‐Cas13a–based nucleic acid detection strategy and was subsequently developed into a mature diagnostic platform that combines isothermal amplification with Cas13a‐mediated collateral cleavage, enabling highly specific detection at ambient temperature with fluorescence or lateral flow readouts (Gootenberg et al. 2017; Kellner et al. 2019). The clinical applicability of SHERLOCK was further demonstrated in 2020, when it was successfully applied to SARS‐CoV‐2 detection with complete concordance to RT‐qPCR (Patchsung et al. 2020). Building on this framework, a range of CRISPR–isothermal amplification platforms rapidly emerged, including Cas12‐based systems such as DETECTR and HOLMES, which couple RPA or LAMP with Cas12a or Cas12b collateral cleavage activity for rapid and sensitive detection (Chen et al. 2018; Li et al. 2018, 2019), as well as Cas13‐centred platforms such as SHINE that enable single‐pot amplification and detection for point‐of‐care applications (Arizti‐Sanz et al. 2020).

Despite these advances, current CRISPR‐based diagnostic platforms still face challenges, including multi‐step workflows, the relatively large size of commonly used Cas effectors and sequence constraints such as protospacer‐flanking site (PFS) or protospacer‐adjacent motif (PAM) requirements. These limitations have stimulated growing interest in Cas13d as a next‐generation CRISPR effector for simplified isothermal diagnostics. Compared with other Cas13 family members, Cas13d retains efficient crRNA‐guided target recognition and collateral RNase activity while featuring a substantially smaller protein size and minimal sequence constraints, making it particularly attractive for portable and integrated diagnostic platforms (Yan et al. 2018; Xue et al. 2022; Yang and Patel 2024). Notably, Konermann et al. purified *Eubacterium siraeum* Cas13d (EsCas13d) based on its robust recombinant expression in *E.coli* and demonstrated that EsCas13d can be activated by target RNA binding to induce collateral cleavage of bystander RNA molecules (Konermann et al. 2018). These characteristics provide the mechanistic basis for integrating EsCas13d with isothermal amplification to achieve rapid, highly specific nucleic acid detection.

Getah virus (GETV) is a mosquito‐borne alphavirus with a broad host range and increasing recognition as an emerging zoonotic pathogen (Azerigyik et al. 2023). The number of disease outbreaks caused by GETV in animals is increasing alongside the types of animals infected, from horses and pigs to cattle, blue foxes and red pandas (Li et al. 2022). Surveillance studies further demonstrate sustained and widespread circulation of GETV in animal populations. In Xinjiang, China, seroprevalence rates reached 74.8% in thoroughbred horses and 51.1% in pigs between 2017 and 2020, indicating intense viral activity at the animal‐mosquito interface (Shi et al. 2022). Similarly, repeated detection of GETV in mosquitoes and racehorses during long‐term surveillance in Japan underscores its capacity for persistence and re‐emergence in endemic areas (Ochi et al. 2023). Together, these findings suggest that GETV circulation in animals and vectors may pose an underrecognized zoonotic risk, underscoring the urgent need for rapid and field‐deployable diagnostic tools to support integrated animal–human surveillance and early outbreak warning.

Here, we report the development of a rapid, one‐step and visual detection (ROSVD) assay for GETV that integrates reverse transcription recombinase‐aided amplification (RT‐RAA) with CRISPR EsCas13d‐mediated collateral RNA cleavage. Notably, ‘one‐step’ in this study does not refer to a single enzymatic reaction occurring simultaneously, but rather a single‐tube, closed‐system workflow. This assay enables sensitive and specific detection of GETV within 30 min at a constant temperature of 37°C, eliminating the need for thermal cycling, complex instrumentation or multi‐step reaction handling. Signal output is directly visualized under UV light, allowing result interpretation without specialized analytical equipment. By combining isothermal amplification, a compact and highly active EsCas13d effector and a closed‐tube workflow, the ROSVD method provides an instrument‐independent and contamination‐resistant diagnostic solution. This approach is particularly well‐suited for on‐farm and field settings, offering a practical tool for rapid GETV surveillance, early outbreak detection and timely disease control.

## 2 Materials and Methods

### 2.1 Virus and Samples

The GETV, PRRSV, PCV 2, PRV, PEDV and PDCoV used in this study were obtained from Sichuan Agricultural University, College of Veterinary Medicine. 56 Clinical samples were obtained from pigs suspected of GETV infection and previously collected in our laboratory.

### 2.2 Viral Lysis and DNA/RNA Extraction

For rapid nucleic acid preparation, clinical samples were processed directly with a Rapid Nucleic Acid Release Reagent (DNA)–Type II (Amplification Future, China), enabling crude nucleic acid release without conventional extraction kits. In parallel, nucleic acids were extracted using the FastPure Viral DNA/RNA Mini Kit V2 (Vazyme, China), followed by reverse transcription with the PrimeScript RT Reagent Kit (Takara, Japan) and used as a reference method for comparison.

### 2.3 Design of Primers, Guide DNA and Reporter RNA

Ten GETV gene sequences were retrieved from GenBank and analysed using MEGA 7.0 to identify conserved regions. Within these regions, three upstream and three downstream RAA primers were designed, yielding nine primer pair combinations for subsequent screening. In parallel, three guide DNAs (gDNAs) targeting conserved sequences within the RAA amplicons were designed for CRISPR EsCas13d‐mediated detection. Two single‐stranded RNA reporters (reporter RNA) were designed: a fluorescence‐based reporter (5′‐6‐FAM‐UUUUUU‐BHQ1‐3′) and a lateral flow immunoassay‐compatible reporter (5′‐6‐FAM‐UUUUUU‐Biotin‐3′). All sequence information was compiled in the supplementary table (Table S1) and synthesized by Sangon Biotech (China).

### 2.4 Expression, Purification of EsCas13d and Buffer Preparation

The pET28a‐MH6‐EsCas13d expression plasmid was purchased from Addgene. In this study, the expression and purification of the EsCas13d protein were performed according to a previously described protocol (Kushawah et al. 2020). The purified protein was verified by Western blot analysis, and the validated protein was stored at −80°C until use.

To ensure smooth progression of the reaction, we prepared a 2× reaction buffer (buffer 1) for two‐step EsCas13d detection with the following composition: 40 mmol/L HEPES (pH 7.1), 100 mmol/L KCl, 10 mmol/L MgCl_2_ and 10% (v/v) glycerol.

### 2.5 RT‐RAA Primers Screening

To screen for the most suitable primer pair, nine primer pairs were evaluated using an RT‐RAA kit (Hangzhou ZC Bio‐Sci & Tech Co. Ltd., China). The reaction mixture was prepared according to the manufacturer's instructions and subjected to isothermal amplification. After amplification, the reaction products were mixed with 6× DNA loading dye and analysed by 1.5% agarose gel electrophoresis. The gel image was captured using a gel documentation system (Bio‐Rad, USA). The corresponding amplification product was purified using the TlANquick Midi Purification Kit (Tiangen, China) according to the manufacturer's protocol and designated as the target DNA.

### 2.6 Target RNA and crRNAs Preparation by in Vitro Transcription

In this study, the target RNA and crRNAs were synthesized by in vitro transcription. A single‐stranded DNA template containing the reverse complementary sequence of the crRNA and a T7 promoter was synthesized. The annealing reaction mixture consisted of 1 μL of gDNA (10 μM), 1 μL of T7 promoter (100 μM), and 8 μL of DNase/RNase‐free water. The mixture was denatured at 95°C for 5 min and then allowed to anneal. Subsequently, in vitro transcription was performed using the T7 High Yield RNA Synthesis Kit (BBI Life Sciences, China) according to the manufacturer's instructions. The purified target DNA and the annealed gDNA were transcribed at 37°C for 4 h to generate the target RNA and crRNA, respectively. The transcribed RNA was purified using a column‐based RNA purification kit (Sangon Biotech, China). The concentration and purity of the resulting target RNA and crRNA were determined using a ScanDrop 100 micro‐volume spectrophotometer (Analytik Jena, Germany), and the final products were stored at −80°C until use.

### 2.7 crRNA Screening

The Cas13d reaction for the crRNA screening was performed using the following conditions: 10 μL buffer 1 (2×), 1 micandidate crRNA 1 μL, 1 μL target RNA, 2 μL reporter RNA (10 nM), 2 μL RNase Inhibitor (BBI Life Sciences, China), 1 μL Cas13d protein and added with DNase/RNase‐free water to 20 μL. Reactions were incubated at 37°C in a real‐time quantitative PCR system, with fluorescence signals recorded at one‐minute intervals over 30 min. Select the crRNA that produces the strongest fluorescent signal for subsequent experiments.

### 2.8 Validation of Cas13d Activity

To verify the activity of the EsCas13d protein, four reaction mixtures were prepared. Reaction mixture 1 contained all components, including 10 μL of buffer 1 (2×), 300 nM crRNA, 1 × 10^6^ pM target RNA, 2 μL of reporter RNA (10 nM), 2 μL of RNase inhibitor (BBI Life Sciences, China), and 600 nM Cas13d protein, with DNase/RNase‐free water added to a final volume of 20 μL. Reaction mixtures 2, 3, and 4 were prepared by replacing Cas13d protein, reporter RNA, or crRNA, respectively, with DNase/RNase‐free water. The reaction mixtures lacking individual components were compared with the complete reaction mixture to assess the contribution of each component.

### 2.9 Construction and Visualization of ROSVD Assay for GETV

To construct a ROSVD assay for GETV, we integrated RT‐RAA, in vitro transcription and CRISPR‐Cas13d detection into a one‐step method based on an original two‐step approach. Due to limited research on the specific buffer composition for the one‐step EsCas13d detection system, this study referred to the SHINE buffer (Arizti‐Sanz et al. 2022), The reaction system consisted of the following components: 1× SHINE buffer, 600 nM EsCas13d protein, 300 nM crRNA, 1 U/μL T7 RNA polymerase, 1 mM rNTPs, 0.5 U/μL RNase inhibitor, 1 μmol/L 5′6‐FAM‐UUUUUU‐BHQ1‐3′ reporter, 25 μM primer F3/R3 and 0.05 U/μL RNase H. The reaction was initiated by adding 14 mM magnesium acetate. After adding the target RNA, the mixture was incubated at 37°C for 30 min. Fluorescence was continuously monitored and recorded every 30 s using a QuantStudio 1 Plus Real‐Time PCR System. Subsequently, the pH and the concentrations of key components (Mg^2+^, KCl and primers) were systematically optimized to adapt the system for one‐step EsCas13d detection. The optimized formulation was designated as buffer A.

For visual detection, we employed three visualization methods. The first method involved incorporating a 5′6‐FAM‐UUUUUU‐BHQ1‐3′ reporter into the reaction system for fluorescence reading using a qPCR instrument. After the reaction (incubated at 37°C or 25°C for 30 min), fluorescence values and amplification curves were recorded by the instrument. In the absence of instrumentation, the reaction tubes could be placed in a 37°C water bath; following a 30‐min incubation, tubes were observed under 470 nm ultraviolet light, with positive samples exhibiting green fluorescence. Alternatively, the 5′6‐FAM‐UUUUUU‐BHQ1‐3′ reporter was replaced with a 5′6‐FAM‐UUUUUU‐Biotin‐3′ reporter. After the reaction, the mixture was applied to a lateral flow assay (LFA) strip, where the appearance of a band at the test line (T) indicated a positive result.

### 2.10 Evaluation of Sensitivity and Specificity

To determine the detection sensitivity of the ROSVD method, serial dilutions of the GETV template were prepared and tested using the ROSVD assay at both 37°C and 25°C, with the latter temperature intended to simulate room‐temperature reactions. To determine the analytical specificity of the ROSVD assay, PRRSV, PCV2, PRV, PEDV and PDCoV were tested as non‐target controls and compared with GETV to verify assay specificity.

### 2.11 Determine the LoD

The limit of detection (LoD) was determined using serial ten‐fold dilutions of target RNA with 20 replicates at each concentration. The LoD was defined as the lowest concentration at which ≥ 95% of replicates yielded positive detection.

### 2.12 Evaluate the Stability of ROSVD Reagents

To evaluate the stability of ROSVD reagents (buffer A), we systematically assessed their performance under room‐temperature storage and repeated freeze–thaw cycles.

### 2.13 The Repeatability and Reproducibility of the ROSVD

The repeatability and reproducibility of the ROSVD assay were evaluated using three concentrations of template (10^6^, 10^4^ and 10^2^ copies/μL). For intra‐assay repeatability, each concentration was tested in six replicates within the same run. For inter‐assay reproducibility, the same samples were tested on three different days using independent reagent preparations. The coefficient of variation (CV) was calculated.

### 2.14 Validation With Clinical Samples

To further assess the clinical applicability of the ROSVD assay, 56 clinical samples suspected of Getah virus infection were collected from Sichuan Province between 2021 and 2025 and analysed using the ROSVD assay. ROSVD results were interpreted using three independent readout modalities. For fluorescence measurement, samples showing fluorescence signals significantly higher than the negative control were considered positive. For visual fluorescence detection, samples exhibiting clearly distinguishable green fluorescence under UV‐light illumination compared with the negative control were interpreted as positive, whereas samples with no visually distinguishable fluorescence were considered negative. For lateral flow strip detection, the appearance of both the test (T) line and the control (C) line was interpreted as a positive result, while the appearance of only the control line indicated a negative result. In parallel, RT‐qPCR was performed on the same samples, and the results of both assays were comparatively evaluated.

### 2.15 Statistical Analysis

Schematic diagrams were created using Adobe Illustrator 2020. Fluorescence signals in the reaction tubes were measured with a fluorescence plate reader (Thermo Fisher Scientific). All data are presented as mean ± standard deviation (SD) from three biological replicates. One‐way analysis of variance (ANOVA) was performed using GraphPad Prism 8.0 software (*p* ≤ 0.05), and graphs were generated accordingly. All figures were organized and finalized using Adobe Illustrator 2020. Graphical abstract image was created with BioGDP.com.

## 3 Results

### 3.1 Workflow of ROSVD

**FIGURE 1:**
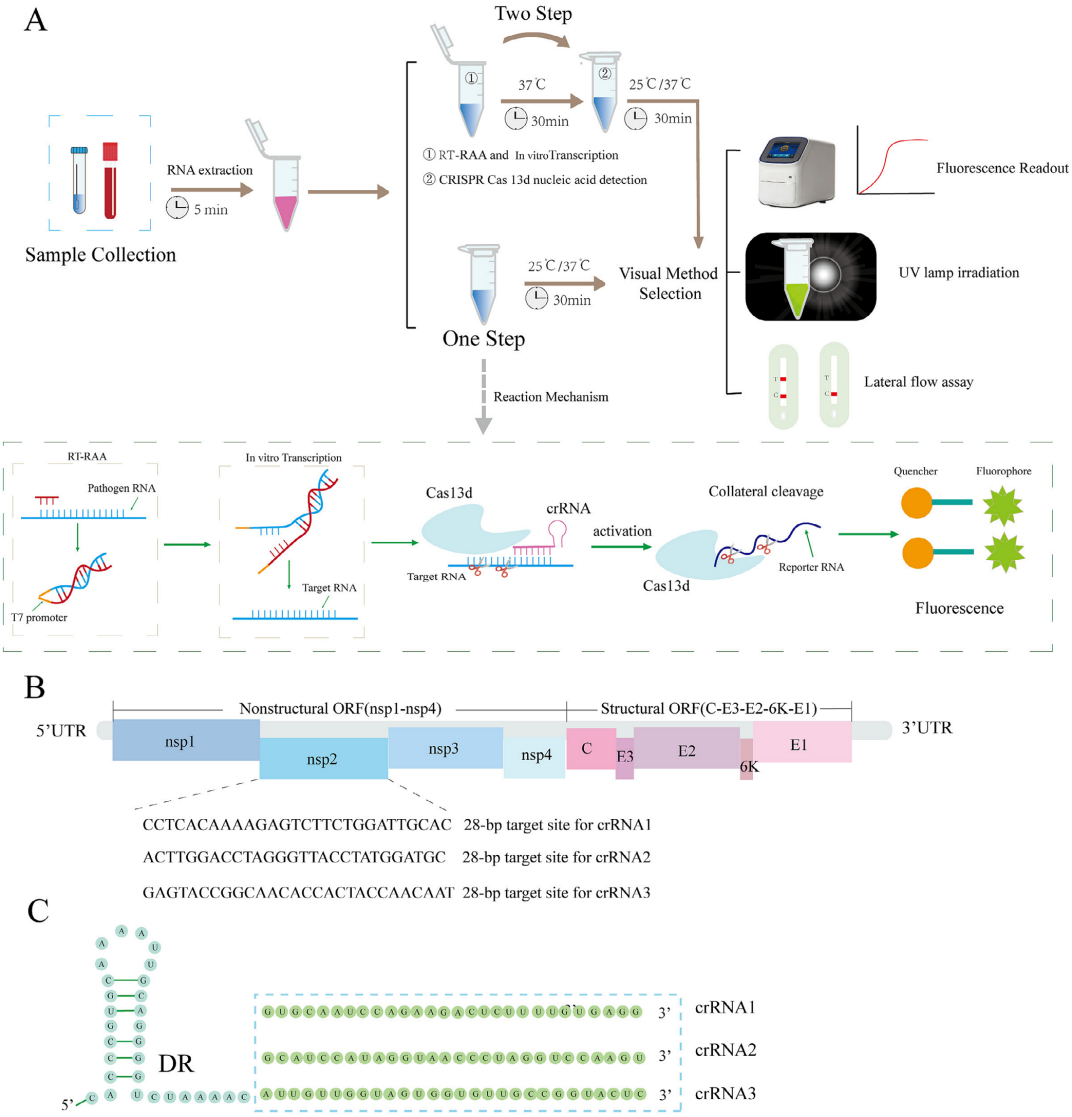
Schematic diagram of the ROSVD platform. (A) The Procedure of the ROSVD Platform. (B) GETV genome diagram. (C) Sequence and Structure of Three Candidate crRNAs. A, B, and C were created by Adobe Illustrator 2020.

In the two‐step method, RT‐RAA and in vitro transcription are performed sequentially, and the main workflow includes RNA extraction, RT‐RAA with in vitro transcription, and CRISPR–EsCas13d‐based nucleic acid detection. In contrast, ROSVD integrates RT‐RAA, in vitro transcription and CRISPR–EsCas13d detection into a single reaction, enabling the entire process to be completed in a single tube. This approach allows one‐step amplification and rapid visual readout of results, thereby simplifying the detection procedure and facilitating on‐site application (Figure 1A). The genomic structure of GETV and the sequence of crRNAs are shown in Figure 1B and Figure 1C.

### 3.2 Validation and Preparation

**FIGURE 2:**
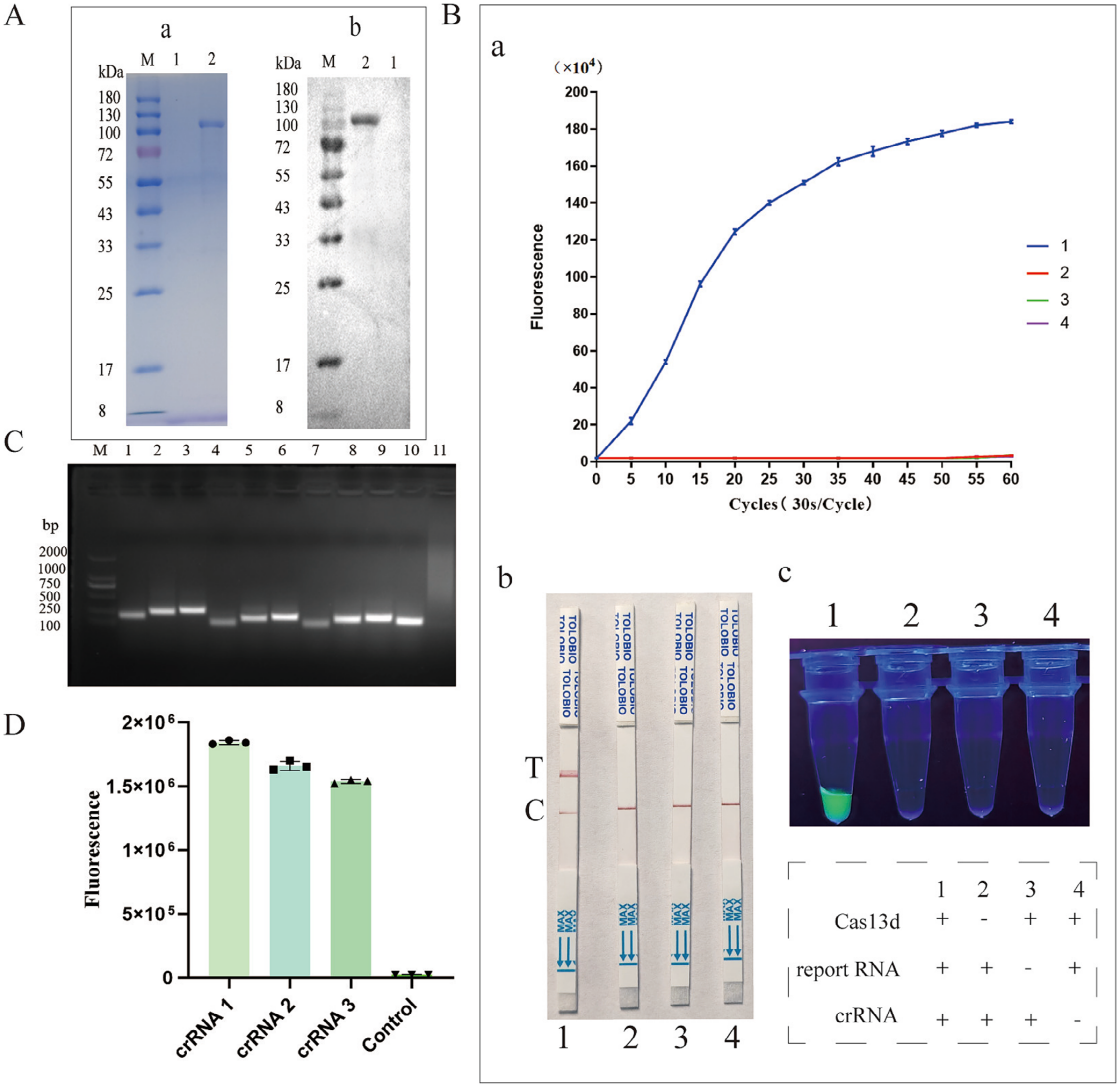
Validation and preparation. (A) Expression and purification of EsCas13d. a: SDS‐PAGE stained with Coomassie blue; b: Western blot; M: Marker; 1: Negative; 2: Cas13d. (B) Verification of Cas13d protein activity. The results in panels a, b, and c were acquired using fluorescence detection, lateral flow assay, and UV light visualization, respectively. (C) RAA primer screening. M: 2000 bp DNA ladder. Lanes 1–9 represent the nine combinations of three forward and three reverse primers: F1‐R1, F1‐R2, F1‐R3, F2‐R1, F2‐R2, F2‐R3, F3‐R1, F3‐R2, F3‐R3. Lane 10: Positive control. Lane 11: Negative control. (D) Screening of crRNAs.

The successful expression and purification of the Cas13d protein (approximately 112.4 kDa) were confirmed by SDS‐PAGE and Western blot analysis (Figure 2A), and its enzymatic activity was subsequently verified. Only the reaction mixture containing all components (Reaction mixture 1) exhibited detectable activity. In contrast, no amplification signal was observed when any of the key components—Cas13d protein, reporter RNA, or crRNA—was omitted from the reaction system, indicating that the purified Cas13d protein retained robust activity (Figure 2B).

Among the nine combinations, F3/R3 demonstrated the highest amplification efficiency (Figure 2C). Therefore, its amplification product was used for subsequent experiments. All three candidate crRNAs (crRNA1, crRNA2 and crRNA3) exhibited strong cleavage activity, with crRNA1 producing the highest fluorescence signal and selected for subsequent analysis (Figure 2D).

### 3.3 Establishment and Optimization of the ROSVD

**FIGURE 3:**
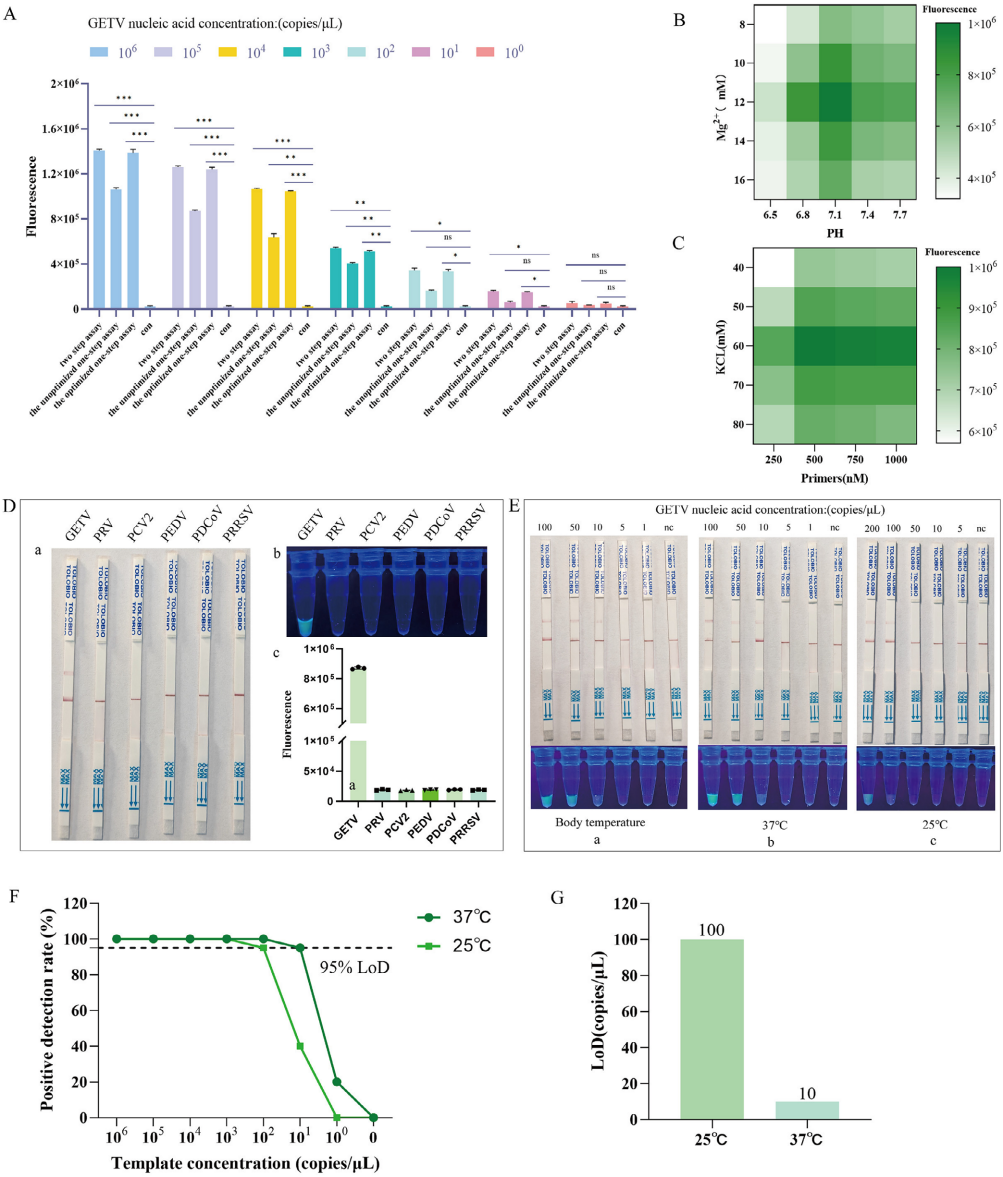
Specificity and Sensitivity of the ROSVD Platform for GETV Detection. (A) Fluorescence values of the two‐step method and the one‐step method (before and after optimization) for detecting different loads of GETV. (B) Optimization of Mg^2+^ concentration and pH in the buffer A; (C) Optimization of KCl and Primers concentration in the buffer A. (D) Specificity of the ROSVD Platform for GETV Detection. The results in panels a, b, and c were acquired using lateral flow assay, UV light visualization, and fluorescence detection, respectively. (E) Sensitivity of the ROSVD Platform for GETV Detection. a, b, and c show the results from reactions conducted at body temperature(approximately 37°C), 37°C and 25°C, respectively. (F) Positive detection rate at different concentrations under 37°C and 25°C. The limit of detection (LoD) was defined as the lowest concentration at which > 95% of replicates yielded positive detection. (G) Summary of LoD under different temperature conditions.

Compared to the two‐step detection method, when using the SHINE buffer (Arizti‐Sanz et al. 2022) for one‐step detection, the sensitivity was reduced (Figure 3A). Therefore, the SHINE buffer is not fully compatible with the CRISPR‐Cas13d‐based one‐step detection assay. We optimized the pH and the concentrations of components (KCl, Mg^2+^ and primers) in the system through iterative adjustments by varying a single parameter at a time. After optimization, the optimal pH for the CRISPR‐Cas13d one‐step detection was found to be 7.1, and the optimal final concentrations of primers, KCl, and Mg^2+^ were 500 nM, 60 mM, and 12 mM, respectively (Figure 3B,C). The sensitivity of the optimized reaction system was comparable to that of the two‐step method (Figure 3A).

The ROSVD assay exhibited excellent diagnostic performance, with a high sensitivity of 100% and a perfect specificity of 100%. The amplification system yielded a signal only with the GETV template and showed no reaction when using PRRSV, PRV, PCV2, PEDV, or PDCoV as templates (Figure 3D). At 37°C, the ROSVD assay demonstrated a detection sensitivity of 10 copies/μL, comparable to that of qPCR. The LoDs at 37°C and 25°C were 10 copies/μL and 100 copies/μL, respectively (Figure 3F,G). Although the analytical sensitivity decreased from 10 copies/μL at 37°C to 100 copies/μL at 25°C (Figure 3E), this level of sensitivity is still expected to be sufficient for detecting most clinically infected animals with moderate‐to‐high viral loads during active outbreaks.

The ROSVD assay showed good repeatability and reproducibility. The intra‐assay CVs ranged from 0.79% to 2.94%, while the inter‐assay CVs ranged from 0.86% to 2.86% (Table S3), indicating high stability and consistency of the assay.

### 3.4 The Utility of the ROSVD Platform for Clinical Detection

**FIGURE 4:**
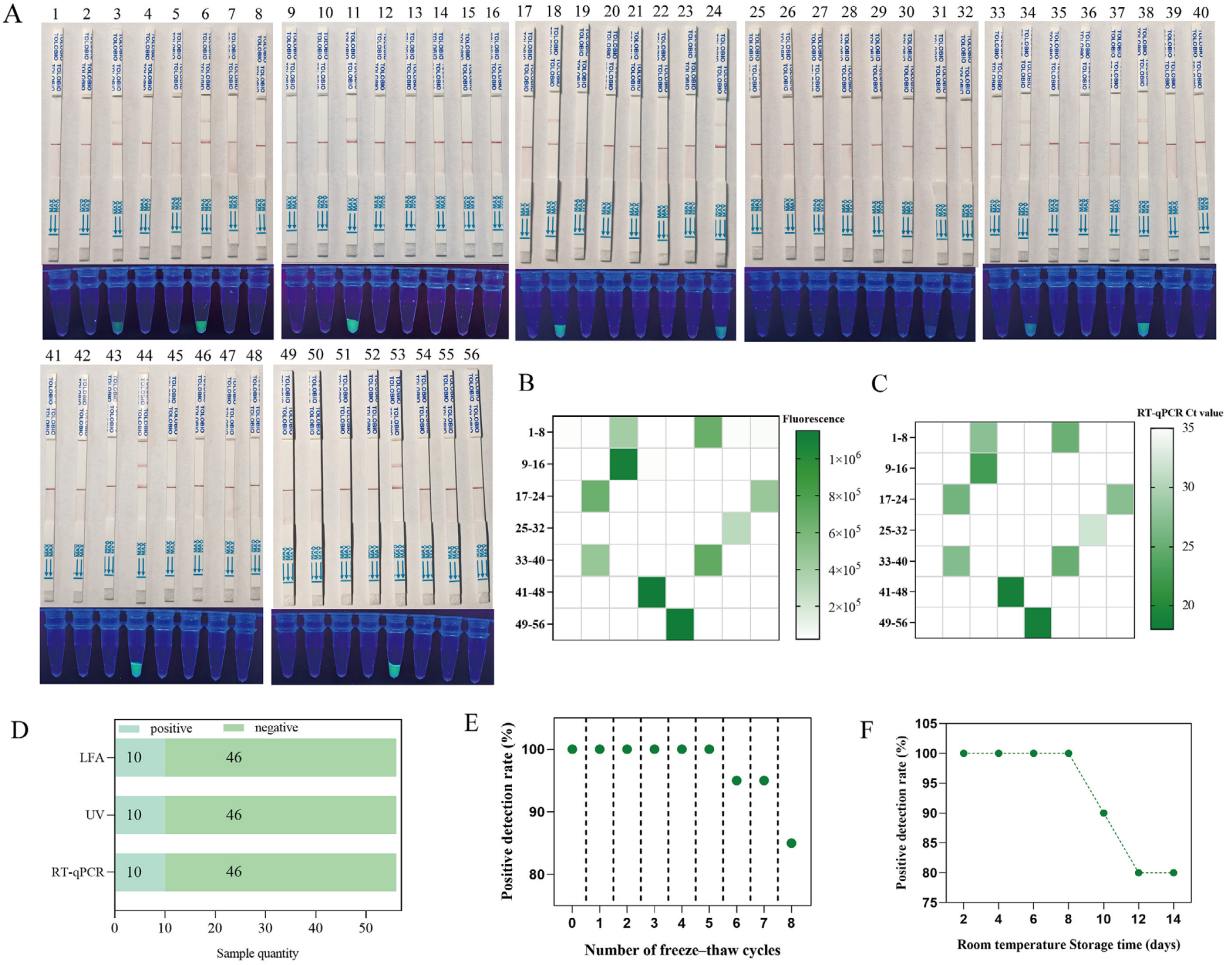
Detection of clinical samples. (A) In‐tube fluorescence and lateral flow assay (LFA) strips for 56 tested samples. (B) Statistical graph of fluorescence values. (C) Statistical graph of Ct value. (D) Statistics of positive and negative results. (E) Detection performance of the ROSVD reagents after repeated freeze–thaw cycles. The detection rate of positive samples was determined following different numbers of freeze–thaw cycles. (F) Room‐temperature storage stability of the ROSVD reagents. The detection rate of positive samples was determined after storage at room temperature for different periods.

To further assess the clinical performance of the ROSVD assay for GETV, we analysed a panel of 56 clinical samples, including 10 positive and 46 negative specimens as determined by the reference RT‐qPCR method. Detailed information about the clinical samples is provided in Table S2. All 11 RT‐qPCR−positive samples were detected by ROSVD via both LFA and in‐tube fluorescence, yielding 100% sensitivity and specificity (Figure 4A–C). The ROSVD assay achieved 100% sensitivity (56/56) (Figure 4D). These results indicate that the ROSVD assay is a highly accurate and reliable tool for detecting clinical pathogens.

### 3.5 The Stability of ROSVD Reagents

To assess the stability of the ROSVD reagents, their detection performance was evaluated following repeated freeze–thaw cycles and storage at room temperature. The detection rate remained at 95% after six freeze–thaw cycles and was still 85% after eight cycles (Figure 4E). Similarly, during room‐temperature storage, the detection rate was maintained at 90% after 10 days and remained at 80% after 14 days (Figure 4F). These results demonstrate that the ROSVD reagents possess good stability under both repeated freeze–thaw conditions and prolonged room‐temperature storage.

## 4 Discussion

GETV circulates widely among animals and, while no human clinical cases have been confirmed, previous serological surveys have detected GETV antibodies in healthy human populations, with some reports indicating seropositivity rates exceeding 10%, suggesting potential zoonotic risk (Yang et al. 2018; Liu et al. 2019; Wang et al. 2022; Zhao et al. 2023). Therefore, effective surveillance calls for diagnostic tools that are both rapid and practical for field use.

The combination of RPA/RAA with CRISPR‐Cas has enabled sensitive detection of pathogens, including SARS‐CoV‐2, HIV and Zika virus (Xue et al. 2022; Zhang et al. 2022), and similar strategies have been applied to animal viruses such as duck circovirus and Nipah virus (Miao et al. 2023; Liang et al. 2024). Yet most of these assays remain two‐step procedures—amplification followed by CRISPR detection—which limits their appeal for on‐site testing. One‐pot systems have emerged recently, but nearly all are built on Cas12a or Cas13a, such as the CRISPRET, which is based on RAA and CRISPR‐Cas13a and offers a simple, field‐applicable, one‐step method for the rapid detection of influenza B virus (Zhang et al. 2025). EsCas13d, despite being the smallest RNA‐targeting effector with strong collateral activity and no PAM/PFS constraints (Yan et al. 2018; Yang and Patel 2024), has received little attention in diagnostic development. In particular, EsCas13d has been reported to exhibit strong and rapid collateral RNase activity, which can enhance signal generation efficiency in fluorescence‐based detection systems (Konermann et al. 2018). In addition, its relatively compact architecture may contribute to faster target‐induced conformational activation, potentially improving reaction kinetics in coupled isothermal amplification–CRISPR detection platforms (Zhang et al. 2018; Liu et al. 2025).

Although one‐pot CRISPR‐based assays have significantly simplified reaction integration by combining amplification and detection into a single reaction, rapid and efficient nucleic acid extraction remains a substantial bottleneck for on‐site nucleic acid detection approaches (Huang et al. 2023). Here we describe ROSVD, a GETV detection method based on CRISPR‐EsCas13d and RT‐RAA that addresses several of these limitations. We combined this workflow with an extraction‐free sample lysis protocol. Reviews on isothermal amplification emphasize that extraction‐free sample processing is essential for true sample‐to‐answer systems (Wilkinson et al. 2024). In this context, the most significant departure from existing one‐pot assays is the incorporation of a simplified nucleic acid release step that works directly on clinical samples, eliminating the need for extraction kits and associated equipment. This extraction‐free design reduces turnaround time and operational complexity while minimizing nucleic acid loss, representing a practical advantage for point‐of‐care testing in settings with limited laboratory infrastructure.

In terms of performance, ROSVD leverages EsCas13d's high collateral activity and RT‐RAA robustness to achieve sensitivity comparable to RT‐qPCR, but without the need for thermal cycling or specialized instrumentation. The assay exhibited LoDs of 10 copies/μL at 37°C and 100 copies/μL at 25°C, indicating that it retained sufficient detection capability under ambient temperature conditions, although with reduced sensitivity compared with operation at 37°C. This level of performance is still suitable for preliminary screening and surveillance in resource‐limited settings where precise temperature control is unavailable. In practical field applications, simple heat sources, such as body heat or portable heating devices, could be used to maintain reaction temperatures near 37°C, thereby improving assay sensitivity without requiring laboratory equipment. Visual readout via UV fluorescence or lateral flow assay further supports field deployment.

Beyond analytical sensitivity, reproducibility and reagent stability are also critical considerations for point‐of‐care molecular diagnostics. In this study, ROSVD exhibited low intra‐assay and inter‐assay variability, demonstrating excellent reproducibility across independent experiments. Furthermore, the reagents maintained stable performance following repeated freeze–thaw cycles and room‐temperature storage, suggesting good resilience during transportation and temporary storage. Together with its extraction‐free workflow and single‐tube design, these characteristics further support the suitability of ROSVD for decentralized and resource‐limited diagnostic settings.

Nevertheless, ROSVD has several limitations. Compared with qPCR‐based assays, ROSVD currently lacks multiplexing capacity and quantitative measurement. However, in the context of rapid screening and field‐based surveillance of GETV, where timely identification is often more critical than absolute quantification, qualitative detection can still provide practical diagnostic value. Future efforts could integrate ROSVD into enclosed or microfluidic platforms to enable automated, multiplexed and contamination‐free detection.

## 5 Conclusion

In conclusion, we developed a CRISPR‐EsCas13d–based assay (ROSVD) for rapid detection of GETV. The integration of RT‐RAA with EsCas13d enables sensitive detection under isothermal conditions, with performance comparable to RT‐qPCR. The simplified sample preparation, based on rapid nucleic acid release, eliminates the need for conventional extraction and reduces assay time. Combined with visual readout formats, the method is well‐suited for field and point‐of‐care applications. This work provides a convenient strategy for GETV detection and may support more effective surveillance.

## Author Contributions

**Liu Baoling:** writing – review and editing, writing – original draft, visualization, validation, methodology, software, data curation. **Xu Tong:** methodology, formal analysis, data curation, writing – review and editing, software. **Shao Lina:** validation, visualization, writing – review and editing, software, investigation. **Jia Qinrui:** visualization, methodology, writing – review and editing, formal analysis. **Xu Zhiwen:** funding acquisition, resources, methodology, supervision, conceptualization. **Zhu Ling:** methodology, resources, project administration, writing – review and editing.

## Funding

This work was supported by National Key Research and Development Program of China during the 14th Five‐Year Plan: Research and Development of Key Technologies for the Prevention and Control of Important Diseases in Wild Animals, 2024YFD1800102. Research and Application of Integrated Prevention, Control and Purification Technologies for Major Swine Epidemic Diseases, 2024YFD1800500. Sichuan Province Major Science and Technology Project for Sichuan Pigs during the 14th Five‐Year Plan Period, 2021ZDZX0010‐3. Sichuan Pig Innovation Team of the National Modern Agricultural Industry Technology System, sccxtd‐2024‐08.

## Ethics Statement

The animal experiments were approved by the Laboratory Animal Management Committee of Sichuan Agricultural University and were conducted in accordance with relevant institutional guidelines and local legislation (Approval No. 20240010). Written informed consent was obtained from the animal owners for their participation in this study. This manuscript does not involve human participants, and no potentially identifiable images or data are included.

## Conflicts of Interest

The authors declare no conflicts of interest.

## Supporting information


**Table S1:** Oligo sequences used in this study.
**Table S2:** The detailed information of the clinical samples.
**Table S3:** Reproducibility evaluation of the ROSVD assay.

## Data Availability

The data that support the findings of this study are available from the corresponding author upon reasonable request.
