# Correction to ‘A Rapid, Field‐Deployable Diagnostic Platform for Getah Virus Based on RT‐RAA and CRISPR EsCas13d’

**DOI:** 10.1111/1751-7915.70464

**Published:** 2026-10-26

Baoling, L. , S. Lina , X. Tong , J. Qinrui , Z. Ling , and X. Zhiwen . 2026. “A Rapid, Field‐Deployable Diagnostic Platform for Getah Virus Based on RT‐RAA and CRISPR EsCas13d.” Microbial Biotechnology 19, no. 8: e70429. 10.1111/1751-7915.70429.PMC1345473842572397

In the published article, the funding information is listed as:

“The National Key Research and Development Program of China during the 14th Five‐Year Plan: Research and Development of Key Technologies for the Prevention and Control of Important Diseases in Wild Animals (2024YFD1800102).”

However, we entered the project number incorrectly during manuscript processing under the funding and acknowledgements section. The correct project number should be: 2024YFD1800200.

We apologize for this error.

