# Supplementary material for: A Rapid, Field‐Deployable Diagnostic Platform for Getah Virus Based on RT‐RAA and CRISPR EsCas13d
Source: Microb Biotechnol. 2026 Aug 9;19(8):e70429. doi: 10.1111/1751-7915.70429 (PMC13454738; doi:10.1111/1751-7915.70429)
Supplement: Supplementary file 1 [file MBT2-19-e70429-s001.docx]

Supplementary Table 1. Oligo sequences used in this study

| **Sequence name** | **Sequence (5’-3’)** |
| --- | --- |
| GETV-RAA-F1 | TAATACGACTCACTATAGGGGGAGAGGGTAGAGTGGTTGCTGAAAAAGAT |
| GETV-RAA-F2 | TAATACGACTCACTATAGGGACCTCACAAAAGAGTCTTCTGGATTGCACC |
| GETV-RAA-F3 | TAATACGACTCACTATAGGGAAAGAGTCTTCTGGATTGCACCTCCGCGGG |
| GETV-RAA-R1 | CTGCATCCATAGGTAACCCTAGGTCCAAGT |
| GETV-RAA-R2 | GTTGGTAGTGGTGTTGCCGGTACTCAGTAT |
| GETV-RAA-R3 | GCATTGAATGGTCGACGCATTGTTGGTAGT |
| gDNA1 | CCTCACAAAAGAGTCTTCTGGATTGCAC*GTTTTAGACCCCTGCAATTTTGCACGGGTG***CCCTATAGTGAGTCGTATTA** |
| gDNA2 | ACTTGGACCTAGGGTTACCTATGGATGC*GTTTTAGACCCCTGCAATTTTGCACGGGTG***CCCTATAGTGAGTCGTATTA** |
| gDNA3 | GAGTACCGGCAACACCACTACCAACAAT*GTTTTAGACCCCTGCAATTTTGCACGGGTG***CCCTATAGTGAGTCGTATTA** |
| crRNA1 | *CACCCGUGCAAAAUUGCAGGGGUCUAAAAC*GUGCAAUCCAGAAGACUCUUUUGUGAGG |
| crRNA2 | *CACCCGUGCAAAAUUGCAGGGGUCUAAAAC*GCAUCCAUAGGUAACCCUAGGUCCAAGU |
| crRNA3 | *CACCCGUGCAAAAUUGCAGGGGUCUAAAAC*AUUGUUGGUAGUGGUGUUGCCGGUACUC |
| Reporter RNA 1 | 6-FAM-UUUUUU-BHQ |
| Reporter RNA 2 | 6-FAM-UUUUUU-Biotin |

Note: FAM (6-carboxyfluorescein) denotes a fluorophore; BHQ (Black Hole Quencher) denotes a quencher; Biotin indicates a biotin label; "UUUUUU" represents six uridine residues used to trigger collateral cleavage by Cas1 3d.

Supplementary Table 2. The detailed information of the clinical samples

| Number | Time of sample collection | Sample Description |
| --- | --- | --- |
| 1 | 2024 | Throat swab |
| 2 | 2024 | Throat swab |
| 3 | 2024 | Anal swab |
| 4 | 2024 | Anal swab |
| 5 | 2024 | Anal swab |
| 6 | 2024 | Serum |
| 7 | 2024 | Serum |
| 8 | 2024 | Blood |
| 9 | 2024 | Anal swab |
| 10 | 2024 | Anal swab |
| 11 | 2024 | Blood |
| 12 | 2024 | Throat swab |
| 13 | 2024 | Blood |
| 14 | 2024 | Throat swab |
| 15 | 2024 | Throat swab |
| 16 | 2024 | Blood |
| 17 | 2024 | Blood |
| 18 | 2024 | Anal swab |
| 19 | 2024 | Serum |
| 20 | 2024 | Serum |
| 21 | 2024 | Anal swab |
| 22 | 2024 | Anal swab |
| 23 | 2024 | Throat swab |
| 24 | 2024 | Serum |
| 25 | 2024 | Throat swab |
| 26 | 2024 | Blood |
| 27 | 2024 | Blood |
| 28 | 2025 | Blood |
| 29 | 2025 | Anal swab |
| 30 | 2025 | Anal swab |
| 31 | 2025 | Anal swab |
| 32 | 2025 | Anal swab |
| 33 | 2025 | Anal swab |
| 34 | 2025 | Throat swab |
| 35 | 2025 | Throat swab |
| 36 | 2025 | Throat swab |
| 37 | 2025 | Throat swab |
| 38 | 2025 | Blood |
| 39 | 2025 | Blood |
| 40 | 2025 | Serum |
| 41 | 2025 | Serum |
| 42 | 2025 | Serum |
| 43 | 2025 | Serum |
| 44 | 2025 | Serum |
| 45 | 2025 | Serum |
| 46 | 2025 | Anal swab |
| 47 | 2025 | Anal swab |
| 48 | 2025 | Anal swab |
| 49 | 2025 | Anal swab |
| 50 | 2025 | Anal swab |
| 51 | 2025 | Blood |
| 52 | 2025 | Blood |
| 53 | 2025 | Blood |
| 54 | 2025 | Throat swab |
| 55 | 2025 | Throat swab |
| 56 | 2025 | Throat swab |

Supplementary Table 3. Reproducibility evaluation of the ROSVD assay

| Template concentration  (copies/uL) | Intra-day CV (%) | Inter-day CV (%) |
| --- | --- | --- |
| 10^6^ | 0.79 | 0.86 |
| 10^4^ | 1.82 | 1.18 |
| 10^2^ | 2.94 | 2.86 |
